# Head-to-head comparison of azvudine and nirmatrelvir/ritonavir for the hospitalized patients with COVID-19: a real-world retrospective cohort study with propensity score matching

**DOI:** 10.3389/fphar.2023.1274294

**Published:** 2023-10-13

**Authors:** An-Hua Wei, Lu Zeng, Lu Wang, Lin Gui, Wen-Ting Zhang, Xue-Peng Gong, Juan Li, Dong Liu

**Affiliations:** Department of Pharmacy, Tongji Hospital, Tongji Medical College, Huazhong University of Science and Technology, Wuhan, China

**Keywords:** COVID-19, azvudine, nirmatrelvir/ritonavir, real-world, effectiveness, safety

## Abstract

**Background:** Nirmatrelvir/ritonavir and azvudine have been approved for the early treatment of COVID-19 in China, however, limited real-world data exists regarding their effectiveness and safety.

**Methods:** We conducted a retrospective cohort study involving the hospitalized COVID-19 patients in China between December 2022 and January 2023. Demographic, clinical, and safety variables were recorded.

**Results:** Among the 6,616 hospitalized COVID-19 patients, we included a total of 725 patients including azvudine recipients (N = 461) and nirmatrelvir/ritonavir (N = 264) recipients after exclusions and propensity score matching (1:2). There was no significant difference in the composite disease progression events between azvudine (98, 21.26%) and nirmatrelvir/ritonavir (72, 27.27%) groups (*p* = 0.066). Azvudine was associated with a significant reduction in secondary outcomes, including the percentage of intensive care unit admission (*p* = 0.038) and the need for invasive mechanical ventilation (*p* = 0.035), while the in-hospital death event did not significantly differ (*p* = 0.991). As for safety outcomes, 33 out of 461 patients (7.16%) in azvudine group and 22 out of 264 patients (8.33%) in nirmatrelvir/ritonavir group experienced drug-related adverse events between the day of admission (*p* = 0.565).

**Conclusion:** In our real-world setting, azvudine treatment demonstrated similar safety compared to nirmatrelvir/ritonavir in hospitalized COVID-19 patients. Additionally, it showed slightly better clinical benefits in this population. However, further confirmation through additional clinical trials is necessary.

## Background

The coronavirus disease 2019 (COVID-19) continues to pose a significant threat to global health. It is crucial to have early and appropriate antiviral agents to treat patients at risk for severe COVID-19 or death (Singh and De Wit, 2022). This is important not only to decrease morbidities and mortalities, but also to restore healthcare capacities and facilitate a return to the new normal (Singh and De Wit, 2022). Currently, antiviral therapy for COVID-19 includes the use of neutralizing monoclonal antibodies (mAbs) and direct antiviral agents (Chen et al., 2023). Neutralizing mAbs specifically target the spike protein of severe acute respiratory syndrome coronavirus 2 (SASR-CoV-2), and their neutralizing activities against viruses and preventing viral entry into human cells contributes to therapeutic effects (Miljanovic et al., 2023). Clinical utilization of either single mAbs or combinations of two or more mAbs has proven effective in reducing the frequency of hospitalization, severe forms of COVID-19, and mortality (Singh and De Wit, 2022; Miljanovic et al., 2023). Direct antiviral agents, on the other hand, are designed to target the viral encoded enzymes essential for viral replication. Specifically, the SARS-CoV-2 3CL protease and RNA-dependent RNA polymerase are two key enzymes, and corresponding three antiviral agents, including remdesivir, nirmatrelvir/ritonavir and molnupiravir were recommended by the World Health Organization (WHO) for patients with mild and moderate COVID-19 (Singh and De Wit, 2022; Murakami et al., 2023). Up to date, the clinical effectiveness of COVID-19 antiviral agents in reduction of hospitalization for those at risk for disease progression fluctuates between 30% and 90% (Singh and De Wit, 2022). However, the current evidence regarding the effectiveness and safety of antiviral agents remains inadequate.

In China, several direct antiviral drugs, including nirmatrelvir/ritonavir, azvudine, remdesivir, lopinavir/ritonavir, and molnupiravir have been approved for the treatment of COVID-19 patients (Singh and De Wit, 2022; Murakami et al., 2023; Mazzitelli et al., 2023b). It is worth noting that, except for remdesivir, which is administered intravenously, the others are oral drugs (Marzi et al., 2022). Among them, oral nirmatrelvir/ritonavir was the first to be granted approval for treating mild to moderate COVID-19 in both adult and paediatric patients who were at high risk of developing severe disease within 5 days of symptom onset. Nirmatrelvir is r is a potent and selective inhibitor of the SARS-CoV-2 3CL protease, while ritonavir is an HIV-1 protease inhibitor and CYP3A inhibitor. By Inhibiting the SARS-CoV-2 3CL protease, viral replication can be prevented by blocking the processing of polyprotein precursors (Hammond et al., 2022; Marzi et al., 2022; Amani and Amani, 2023). Up to date, numerous clinical trials and real-world studies have been conducted to evaluate the effectiveness and safety of nirmatrelvir/ritonavir. The majority of these studies have consistently shown that nirmatrelvir/ritonavir significantly reduces the severity of COVID-19 and mortality (Wong et al., 2022a; Wong et al., 2022b; Wen et al., 2022; Zhou et al., 2022; Cheema et al., 2023; Zheng et al., 2023). Azvudine, on the other hand, is the first double-target nucleoside drug and has demonstrated significant and broad-spectrum antiviral effects *in vitro* (Wang et al., 2014; Zhang et al., 2021). An phase three multicenter randomized clinical study further suggested that azvudine significantly shorten the symptom improvement time and increase the proportion of mild and common COVID-19 patients with improved clinical symptoms (Yu and Chang, 2022). Real-world studies have also confirmed the substantial clinical benefits of azvudine treatment in hospitalized COVID-19 patients (Ren et al., 2020; Shen et al., 2023). As a result, the National Medical Products Administration (NMPA) granted conditional authorization for the use of azvudine in the treatment of COVID-19 on 25 July 2022. In China, both nirmatrelvir/ritonavir and azvudine were approved by the National Healthcare Security Administration on 12 August 2022 for inclusion in the medical reimbursement list.

While current guidelines prioritize the use of direct antiviral drugs in COVID-19 patients, there is still a need for more clinical data on their real-world effectiveness and safety. In this retrospective cohort study, we aimed to conduct a head-to-head comparison of the clinical effectiveness and safety of nirmatrelvir/ritonavir and azvudine in hospitalized COVID-19 patients at the Tongji Hospital, as the largest hospital in the central region of China and the main treatment facility for acute or critical COVID-19 patients during a specific pandemic wave.

## Methods

### Patient population and data elements

We conducted a single-center, retrospective cohort study involving the hospitalized adult patients (aged ≥18 years) with COVID-19 (confirmed by RT-PCR), who were given azvudine or nirmatrelvir/ritonavir plus standard treatment at Tongji hospital of Huazhong University of Science and Technology, during the period from 1 Dec 2022 to 31 Jan 2023. This study was approved by the institutional review board of Tongji hospital (TJ-IRB20230202). Patient data were extracted from the hospital’s Electronic Medical Records (EMRs) and anonymized to ensure patient privacy. The EMRs information including demographic characteristics, admission data, diagnoses, clinical categories, prescription and drug dispensing records, procedures, laboratory tests, and discharge or death dates were analyzed. The different clinical categories of COVID-19 were defined according to the Chinese Diagnosis and Treatment Program for Novel Coronavirus Pneumonia (10th Edition). We also considered comorbidities such as diabetes mellitus, cancer, hypertension, cardiovascular disease, cerebral infarction, chronic kidney disease, chronic obstructive pulmonary disease, and chronic liver disease. Additionally, we evaluated the impact of co-medications, including baricitinib, systemic steroid and tocilizumab. Baseline laboratory parameters and changes in values over time were collected from the EMRs, encompassing complete blood cell count, electrolyte levels, renal function, hepatic function, and coagulation function.

### Outcome definition

The primary outcome of our study was defined as a composite of disease progression events, including the intensive care unit admission, the need for invasive mechanical ventilation, and in-hospital death. Additionally, we also analyzed each of these events individually as secondary outcomes. In terms of safety outcomes, we assessed the incidence of adverse events and categorized them based on various organ systems.

### Propensity matching

To account for potential confounding factors, propensity score (PS) models were employed in our study. Baseline covariates and laboratory parameters on admission of patients, such as age, gender, BMI, comorbidities, severity of COVID-19 on admission, concomitant treatments initiated at admission were included to be analyzed. We used PS models conditional on the aforementioned baseline covariates, which was performed with a 1:2 match between two groups with a calliper width of 0.02 without replacement. All baseline variables in the PS-matched cohort were descriptively analyzed, and then the standard mean differences (SMDs) were used to assess the balance of each baseline covariate between the groups before and after PS- matching. Subgroup analyses were performed at each level of the baseline covariates above to assess the robustness of the estimates.

### Statistical analysis

Quantitative variables were summarized using medians with interquartile ranges (IQRs), while qualitative variables were presented as absolute and percentage frequencies. Baseline characteristics were compared between patients using appropriate statistical tests. Student’s *t*-test for near-normal continuous variables, the Mann-Whitney *U*-test for other continuous variables, and the chi-square test (or Fisher’s exact test when appropriate) for categorical variable. Missing data were not imputed for any of the baseline variables. All *p* values were two-sided and *p* < 0.05 was considered statistically significant. The statistical analyses were performed using R version 3.6.3 and python version 3.7.

## Results

### Patient characteristics

**FIGURE 1:**
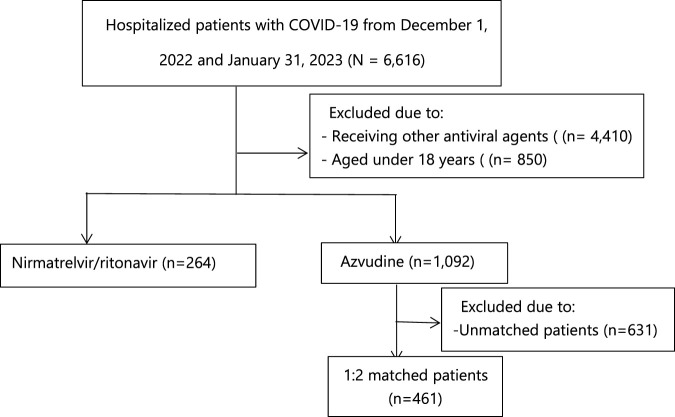
Identification of azvudine recipients and nirmatrelvir-ritonavir recipients among the hospitalized COVID-19 patients.

As shown in Figure 1, a total of 6,616 patients with COVID-19 were admitted to Tongji Hospital. After excluding patients under 18 years old and those who receiving other treatments, the final database included 1,356 patients, with 1,092 in azvudine group and 264 in nirmatrelvir/ritonavir group. Supplementary Table S1 provides details of the missing baseline laboratory data. Table 1 presents the baseline characteristics of the 1,356 patients before PS-matching. Patients in azvudine group were older [median age 65 (IQR, 54–77) vs. 70 (60–77) years, *p* = 0.004], but there were no significant differences in terms of sex (*p* = 0.943) and BMI (*p* = 0.108). The azvudine group had a lower proportion of patients with chronic kidney disease (14.74% vs. 20.83%, *p* = 0.015), and a higher proportion with cardiovascular disease (17.86% vs. 10.23%, *p* = 0.003) and hypertension (33.61% vs. 11.74%, *p* < 0.001). A lower proportion of patients with moderate clinical categories on admission (25.64% vs. 41.67%, *p* < 0.001) and receiving systemic steroids (62.64% vs. 82.58%, *p* < 0.001) was observed in azvudine group. Other comorbidities (diabetes mellitus, cancer, cerebral infarction, chronic obstructive pulmonary disease, and chronic liver disease) and co-medications (baricitinib and tocilizumab) did not significantly differ between the two groups. In terms of laboratory parameters, there were significant differences in the values of red blood cell count (RBC), hemoglobin (Hg), platelet count (PLT), and total bilirubin (TB). Patients treated with nirmatrelvir/ritonavir had significantly lower values of RBC (3.78 ± 0.89 vs. 3.99 ± 0.72 *10^12^/L, *p* < 0.001), Hg (114.11 ± 26.15 vs. 121.61 ± 21.35 g/L, *p* < 0.001), PLT (181.03 ± 106.75 vs. 221.89 ± 249.30) *10^9^/L, *p* = 0.01), and TB (9.66 ± 6.52 vs. 10.68 ± 7.31 umol/L, *p* = 0.04), whereas there were no significant difference in values of white blood cell count (WBC), absolute neutrophil count (NEU), neutrophil percentage (NEUP), aspartate transaminase (AST), alanine transaminase (ALT), alkaline phosphatase (ALP), lactate dehydrogenase (LDH), estimated glomerular filtration rate (eGFR), serum creatinine (CCR), urea (U), uric acid (UA), sodium (NA), potassium (K), chloride (CL), Thrombin time (TT), fibrinogen (FBG), activation of partial thromboplastin time (APTT), and prothrombin time (PT). After 1:2 propensity score matching, a total of 725 patients were included to be analysis. Supplementary Figure S1 shows the distributions of covariates before and after PS matching. As shown in Table 1, demographics and comorbidities did not significantly differ between the PS-matched groups.

**TABLE 1:** Baseline characteristics of the participants before and after propensity score matching.

	unmatched (*n* = 1356)			matched (*n* = 725)		
Characteristics	Azvudine (*n* = 1092)	nirmatrelvir-ritonavir (*n* = 264)	*p*	Azvudine (*n* = 461)	nirmatrelvir-ritonavir (*n* = 264)	*p*
Gender,n(%)			0.943			0.754
Male	378 (34.62)	92 (34.85)		295 (63.99)	172 (65.15)	
Female	714 (65.39)	172 (65.15)		166 (36.01)	92 (34.85)	
BMI(kg/m^2^), mean(±SD)	24.04 ± 3.74	23.54 ± 3.60	0.108	23.67 ± 3.80	23.54 ± 3.60	0.713
Age(yr), median[IQR]	70 [60,77]	65 [54,77]	0.004	68 [57,76]	65 [54,77]	0.389
Comorbidities, n(%)						
Diabetes mellitus	292 (26.74)	66 (25.00)	0.565	111 (24.08)	66 (25.00)	0.781
Cancer	135 (12.36)	44 (16.67)	0.064	75 (16.27)	44 (16.67)	0.889
Hypertension	367 (33.61)	31 (11.74)	<0.001	61 (13.23)	31 (11.74)	0.562
Cardiovascular disease	195 (17.86)	27 (10.23)	0.003	71 (15.40)	27 (10.23)	0.053
Cerebral infarction	112 (10.26)	21 (7.96)	0.259	38 (8.24)	21 (7.96)	0.891
Chronic kidney disease	161 (14.74)	55 (20.83)	0.015	93 (20.17)	55 (20.83)	0.832
Chronic obstructive pulmonary disease	39 (3.57)	7 (2.65)	0.459	14 (3.04)	7 (2.65)	0.766
Chronic liver disease	36 (3.30)	7 (2.65)	0.591	16 (3.47)	7 (2.65)	0.545
Clinical categories, n (%)			<0.001			0.363
Moderate	280 (25.64)	110 (41.67)		129 (27.98)	87 (32.96)	
Severe	456 (41.76)	92 (34.85)		217 (47.07)	114 (43.18)	
Critical	356 (32.60)	62 (23.49)		115 (24.94)	63 (23.86)	
Co-medications, n(%)						
Baricitinib,n (%)	14 (1.28)	8 (3.03)	0.044	8 (1.74)	8 (3.03)	0.253
Systemic steroid, n (%)	684 (62.64)	218 (82.58)	<0.001	370 (80.26)	218 (82.58)	0.443
Tocilizumab, n (%)	10 (0.92)	7 (2.65)	0.023	7 (1.52)	7 (2.65)	0.286
Laboratory maker, mean(±SD)						
RBC(*10^12^/L)	3.99 ± 0.72	3.78 ± 0.89	<0.001	3.88 ± 0.77	3.78 ± 0.89	0.138
WBC(*10^9^/L)	7.45 ± 5.48	8.20 ± 26.28	0.391	7.00 ± 6.00	6.62 ± 4.43	0.38
Hg (g/L)	121.61 ± 21.35	114.11 ± 26.15	<0.001	117.34 ± 22.21	114.22 ± 26.02	0.106
PLT (*10^9^/L)	221.89 ± 249.30	181.03 ± 106.75	0.01	193.78 ± 93.27	181.68 ± 106.32	0.115
NEU(*10^9^/L)	5.81 ± 4.15	6.63 ± 23.29	0.572	5.49 ± 3.79	6.63 ± 23.29	0.308
NEUP(%)	75.59 ± 14.60	74.25 ± 15.89	0.192	74.96 ± 15.14	74.26 ± 15.98	0.568
AST (U/L)	48.97 ± 209.26	39.53 ± 85.40	0.476	53.99 ± 283.75	39.68 ± 86.05	0.433
ALT (U/L)	34.79 ± 82.00	34.12 ± 88.77	0.907	36.76 ± 115.66	33.76 ± 89.24	0.722
ALP(U/L)	81.00 ± 47.45	82.61 ± 43.77	0.618	81.69 ± 45.06	82.11 ± 43.59	0.905
LDH(U/L)	322.37 ± 184.24	303.80 ± 142.07	0.129	315.41 ± 174.09	300.92 ± 127.94	0.248
TB (umol/L)	10.68 ± 7.31	9.66 ± 6.52	0.04	10.26 ± 7.30	9.66 ± 6.52	0.278
eGFR (mL/min/1.73m^2^)	71.05 ± 28.16	71.96 ± 31.40	0.674	70.09 ± 30.24	71.31 ± 31.15	0.617
CCR(umol/L)	130.08 ± 196.79	125.35 ± 141.48	0.715	144.36 ± 240.60	125.35 ± 141.48	0.246
U (mmol/L)	8.92 ± 7.72	8.79 ± 7.19	0.815	9.41 ± 8.90	8.87 ± 7.22	0.41
UA (mmol/L)	295.89 ± 138.53	292.77 ± 123.80	0.74	296.27 ± 143.61	293.28 ± 124.49	0.782
NA (mmol/L)	137.16 ± 4.93	136.91 ± 4.48	0.466	137.23 ± 4.19	136.88 ± 4.49	0.305
K (mmol/L)	4.16 ± 0.60	4.20 ± 0.55	0.299	4.19 ± 0.63	4.21 ± 0.55	0.626
CL (mmol/L)	101.62 ± 5.25	101.56 ± 4.83	0.882	101.71 ± 4.60	101.54 ± 4.86	0.647
TT(s)	18.32 ± 10.28	17.76 ± 2.03	0.376	17.92 ± 3.21	17.76 ± 2.04	0.428
FBG (g/L)	4.69 ± 1.38	4.65 ± 1.42	0.643	4.58 ± 1.40	4.65 ± 1.43	0.51
APTT(s)	37.65 ± 9.82	38.19 ± 7.24	0.408	38.49 ± 10.81	38.18 ± 7.28	0.686
PT(s)	13.53 ± 2.45	13.50 ± 1.75	0.818	13.66 ± 3.05	13.49 ± 1.75	0.42

### Clinical outcomes of PS-matched cohort

Table 2 presents the clinical and safety outcomes observed in the PS-matched cohort. The composite outcome did not show a significant difference between azvudine and nirmatrelvir/ritonavir groups (*p* = 0.066). The composite disease progression events occurred in 98 (21.26%) patients treated with azvudine and 72 (27.27%) patients treated with nirmatrelvir/ritonavir. However, we did observe a significant reduction in secondary outcome measures associated with azvudine treatment, including the percentage of intensive care unit admission (*p* = 0.038) and the need for invasive mechanical ventilation (*p* = 0.035), while the event of in-hospital death did not significantly differ (*p* = 0.991).

**TABLE 2:** Clinical effectiveness and safety outcomes among azvudine *versus* nirmatrelvir-ritonavir recipients.

Outcomes	Azvudine (*n* = 461)	Nirmatrelvir-ritonavir (*n* = 264)	*p*-value
Primary outcome	98 (21.26)	72 (27.27)	0.066
Secondary outcomes			
Intensive care unit admission	11(2.39)	14(5.30)	0.038
Need for invasive mechanical ventilation	77(16.70)	61(23.11)	0.035
In-hospital death	63 (13.67)	36 (13.64)	0.991
Safety outcome			
All drug-related adverse events	33 (7.16)	22 (8.33)	0.565
Change of laboratory maker, mean(±SD)			
RBC(*10^12^/L)	0.36 ± 0.36	0.38 ± 0.33	0.541
WBC(*10^9^/L)	6.52 ± 8.74	7.80 ± 11.79	0.152
Hemoglobin (g/L)	10.84 ± 9.99	11.83 ± 10.07	0.374
Platelet count (*10^9^/L)	115.56 ± 297.07	120.49 ± 352.49	0.866
NEU (*10^9^/L)	5.76 ± 5.84	6.82 ± 6.72	0.058
NEUP(%)	13.44 ± 12.61	14.75 ± 13.05	0.276
AST (U/L)	100.67 ± 516.37	173.67 ± 759.55	0.301
ALT (U/L)	43.34 ± 105.89	72.14 ± 174.16	0.056
ALP(U/L)	38.79 ± 56.84	60.45 ± 144.61	0.052
LDH(U/L)	205.38 ± 319.45	214.42 ± 324.78	0.798
TB (umol/L)	7.87 ± 14.93	8.41 ± 12.76	0.714
eGFR (mL/min/1.73m^2^)	13.81 ± 14.07	17.76 ± 17.65	0.007
CCR(umol/L)	61.61 ± 114.77	62.82 ± 105.91	0.926
U (mmol/L)	6.79 ± 10.49	8.12 ± 12.05	0.213
UA (mmol/L)	110.59 ± 142.35	100.85 ± 102.08	0.501
NA (mmol/L)	6.25 ± 5.66	7.12 ± 6.09	0.094
K (mmol/L)	0.70 ± 0.69	0.79 ± 0.65	0.184
CL (mmol/L)	6.267 ± 5.36	6.96 ± 5.84	0.185
TT(s)	7.56 ± 30.40	19.92 ± 60.32	0.028
FBG (g/L)	1.22 ± 1.12	1.21 ± 1.03	0.926
APTT(s)	9.78 ± 17.40	22.01 ± 48.67	0.021
PT(s)	3.93 ± 8.36	3.46 ± 7.94	0.616
Percentage of eGFR Change			
Decline of eGFR>10%	205 (44.47)	132 (50.00)	0.151
Decline of eGFR>30%	101 (21.91)	64 (24.24)	0.471
Decline of eGFR>50%	63 (13.66)	40 (15.15)	0.581

As for safety outcomes, a total of 33 out of 461 patients (7.16%) in azvudine group and 22 out of 264 patients (8.33%) in nirmatrelvir/ritonavir group were observed the drug-related adverse events during their hospital stay. There was no significant difference between two groups (*p* = 0.565). The most common adverse events in azvudine group were constipation (9), aypnia (7), dizziness (4), diarrhea (4), stomachache (3), headache (3), vomiting (2), drowsiness (1), nausea (1), and melena (1). In the nirmatrelvir/ritonavir group, a total of 22 patients experienced drug-related adverse events, including diarrhea (3), abnormal urination (2), aypnia (2), edema (2), eruption (2), dysphoria (2), stomachache (1), fever (1), feeble (1), arrhythmia (1), bleeding (1), and headache (1). Meanwhile, the change values of laboratory parameters (Table 3), such as the blood cell count, electrolytes, renal function, hepatic function, and coagulation function did not significantly differ between the groups, except for eGFR (*p* = 0.007). In further analysis, no significant difference was observed in the percentage of eGFR decline greater than 10% (*p* = 0.151), 30% (*p* = 0.471) or 50% (*p* = 0.581).

**TABLE 3:** Subgroup analysis of clinical effectiveness and safety outcomes.

Outcomes	Covariates	Subgroup	N	OR	95%CI	*p*-value
Composite disease progression outcome	Overall		725	1.102	[0.743,1.634]	0.629
	Diabetes mellitus	No	548	0.845	[0.531,1.345]	0.478
		Yes	177	2.404	[1.095,5.277]	0.029
Intensive care unit admission	Overall		725	2.291	[1.025,5.122]	0.043
	Cancer	No	606	2.631	[1.106,6.258]	0.029
		Yes	119	0.849	[0.075,9.640]	0.895
	Chronic kidney disease	No	577	1.611	[0.644,4.030]	0.308
		Yes	148	9.2	[1.046,80.937]	0.045
Need for invasive mechanical ventilation	Overall		725	1.499	[1.028,2.184]	0.035
	Diabetes mellitus	No	548	1.199	[0.770,1.869]	0.422
		Yes	177	2.771	[1.321,5.812]	0.007
	Cardiovascular disease	No	627	1.823	[1.189,2.794]	0.006
		Yes	98	1.018	[0.406,2.550]	0.969
	Chronic obstructive pulmonary disease	No	704	1.502	[1.024,2.203]	0.037
		Yes	21	1.467	[0.184,11.718]	0.718
	Clinical categories	Moderate	216	2.89	[1.254,6.661]	0.013
		Severe	331	1.516	[0.837,2.749]	0.17
		Critical	178	1.097	[0.570,2.113]	0.78ll1
	Tocilizumab	No	711	1.607	[1.093,2.364]	0.016
		Yes	14	0.067	[0.005,0.970]	0.047
	Baricitinib	No	709	1.509	[1.030,2.212]	0.035
		Yes	16	1	[0.104,9.614]	1

OR, odds ratio; CI, confidence interval.

The subgroup analyses of outcomes were performed, and the results were presented in Table 3 and Supplementary Tables S2–S6. Patients with diabetes in azvudine group had a lower risk of composite disease progression events compared to the nirmatrelvir/ritonavir group [OR = 2.404, 95%CI (1.095, 5.277), *p* = 0.029]. However, no significant differences were observed in other subgroups. A similar lower risk of needing invasive mechanical ventilation for patients with diabetes in the azvudine group was also found [OR = 2.771, 95%CI (1.321, 5.812), *p* = 0.007]. Greater benefits associated with azvudine treatment were observed in patients with a moderate clinical category on admission, those without concomitant cardiovascular disease and chronic obstructive pulmonary disease, and those who did not receive tocilizumab and baricitinib. Additionally, patients with chronic kidney disease [OR = 9.20, 95%CI (1.046, 80.937), *p* = 0.045] or without cancer [OR = 2.631, 95%CI (1.106, 6.258), *p* = 0.029] had a relatively lower incidence of intensive care unit admission in azvudine group.

## Discussion

To the best of our knowledge, this retrospective cohort study is the first to directly compare both clinical effectiveness and safety of these oral antiviral agents in China. There was no statistically significant difference in the clinical effectiveness, in terms of reducing disease progression, and safety between nirmatrelvir/ritonavir and azvudine in hospitalized COVID-19 patients. However, azvudine showed potential clinical benefits in secondary outcomes for specific subgroups, including patients with diabetes, chronic kidney disease, and those with a moderate clinical category on admission. It is important to note that these findings should be further confirmed through clinical trials with larger sample sizes to establish more robust evidence.

Our findings regarding the clinical effectiveness differ from a previous study conducted at Xiangya Hospital in China (Deng et al., 2023). Deng et al. observed a lower incidence rate of composite disease progression outcome and all-cause death in azvudine recipients, especially in patients aged <65 years, those with a history of disease, those with severe COVID-19 at admission, and those receiving antibiotics. The whole conclusion suggests that azvudine treatment may be more effective compared to nirmatrelvir/ritonavir in terms of composite disease progression outcome. However, our study did not find a significant difference in the composite disease progression outcome between azvudine and nirmatrelvir/ritonavir, except for certain subgroups such as patients with diabetes. It is known that nirmatrelvir/ritonavir has been reported to reduce hospitalization rate and mortality by 88% when initiated within 5 days of symptom onset in high-risk patients in a phase III clinical trial, leading to its authorization for the treatment of high-risk patients with mild to moderate COVID-19 (Arbel et al., 2022). A real-world study conducted in China showed that patients who received nirmatrelvir/ritonavir had more rapid virus suppression in the early stages of hospitalization compared to those who received azvudine (Gao et al., 2023). On the other hand, azvudine was mainly approved to treat all patients with common and severe COVID-19 (Gentile et al., 2022). Though the results of the phase III study have not been officially released, both of Shen et al. (2023) and Deng et al. (2023) suggested that azvudine treatment is associated with significantly lower risks of composite disease progression outcome and all-cause death in real-world studies. Therefore, we tried to speculate that initiating treatment with nirmatrelvir/ritonavir as early as possible may provide clinical benefits, while azvudine with a longer course (14 days) of antiviral treatment appears to yield a better therapeutic effect for specific COVID-19 patients. However, there is no doubt that further studies with larger sample sizes are needed to validate these findings. In addition to assessing the composite disease progression outcome, our study also examined other disease progression indicators, such as in-hospital death, admission to the intensive care unit, and the need for invasive mechanical ventilation. We observed a relative advantage of azvudine over nirmatrelvir/ritonavir in terms of the proportion of patients admitted to the intensive care unit and the need for invasive mechanical ventilation.

In our study, the safety of both azvudine and nirmatrelvir/ritonavir was also analyzed. The total incidences of adverse events in azvudine and nirmatrelvir/ritonavir groups were 7.16% and 8.33%, respectively. No serious adverse events were observed, and there was no significant difference in the change values of laboratory parameters. These findings suggest that both azvudine and nirmatrelvir/ritonavir have an overall favorable safety profile for the treatment of COVID-19 patients, which aligns with the results of a recent meta-analysis involving 2,143 patients (Amani and Amani, 2023). From the incidence of adverse events perspective, our data, albeit limited, suggested an overall lower frequency of adverse events compared to clinical trials or other cohort studies (Gentile et al., 2022; Amani and Amani, 2023; Mazzitelli et al., 2023a; Cheema et al., 2023; Gao et al., 2023). For example, in a phase 2–3 double-blind, randomized, controlled trial, the incidence of any adverse events during the treatment period was higher with nirmatrelvir/ritonavir compared to placebo (22.6% vs. 23.9%), while serious adverse events were 1.6% vs. 6.6% (Amani and Amani, 2023). Dysgeusia (5.6% vs. 0.3%) and diarrhea (3.1% vs. 1.6%) occurred more frequently with nirmatrelvir/ritonavir than with placebo (Miljanovic et al., 2023). Another real-life study reported adverse events in 12.1% of patients receiving nirmatrelvir/ritonavir treatment, mainly dysgeusia, diarrhea, and nausea. When compared with molnupiravir, 19.1% of patients experienced adverse events following nirmatrelvir/ritonavir intake (Najjar-Debbiny et al., 2023). In our study, the most common adverse events in azvudine group were related to gastrointestinal disorders, psychiatric/nervous system disorders, including constipation, aypnia, dizziness, diarrhea, stomachache, and headache, which was basically in accordance with the instructions and clinical trials. Adverse events associated with nirmatrelvir/ritonavir included diarrhea, abnormal urination, aypnia, edema, eruption, and dysphoria. We observed a potential influence on renal function, as measured by value of eGFR, which appeared to be higher in the nirmatrelvir/ritonavir group compared to the azvudine group. However, there was no significant difference in further analysis. It's important to note that ritonavir, as a strong CYP3A4 inhibitor, may affect the metabolism of various drugs used for arrhythmia, diabetes, and neurological diseases, even when nirmatrelvir/ritonavir is used for a short duration (Loos et al., 2022). Therefore, more clinical trials are needed to investigate in further. Overall, the safety profiles of both azvudine and nirmatrelvir/ritonavir were good, and no serious drug-related adverse events were observed.

There were several limitations in our study. Firstly, the included the hospitalized COVID-19 patients were mainly from Hubei province in China during a specific pandemic wave, thus the results may only be representative of this specific population and cannot be generalized to all COVID-19 patients or other countries. Secondly, the timing of symptom onset and vaccination status were not recorded for some missing data, and the relatively small sample size might have influenced the statistical power of our subgroup analyses. Thirdly, despite our efforts to collect data consecutively and adjust for a wide range of confounders using the propensity score model, we can not completely rule out the possibility of selection bias or confounding by indication in this retrospective cohort study. Randomized controlled trials would provide more rigorous evidence in this regard. Lastly, the incidence of adverse events was determined by pharmacists based on causal criteria, which it subjective to some extent, and there were inevitably the possible of omissions and misstatements for retrospective study. Future prospective studies with standardized protocols for adverse event assessment would provide more accurate and reliable data.

## Conclusion

In conclusion, our study indicates that both azvudine and nirmatrelvir/ritonavir have comparable safety profiles in hospitalized COVID-19 patients. Azvudine showed slightly better clinical benefits in this population, although further clinical trials are necessary to confirm these findings. It is important to continue research efforts to gather more evidence and validate these results obtained in our study.

## Data Availability

The original contributions presented in the study are included in the article/Supplementary Material, further inquiries can be directed to the corresponding authors.
