# Supplementary material for: Head-to-head comparison of azvudine and nirmatrelvir/ritonavir for the hospitalized patients with COVID-19: a real-world retrospective cohort study with propensity score matching
Source: Front Pharmacol. 2023 Oct 13;14:1274294. doi: 10.3389/fphar.2023.1274294 (PMC10603265; doi:10.3389/fphar.2023.1274294)
Supplement: Supplementary file 1 [file DataSheet1.docx]

**
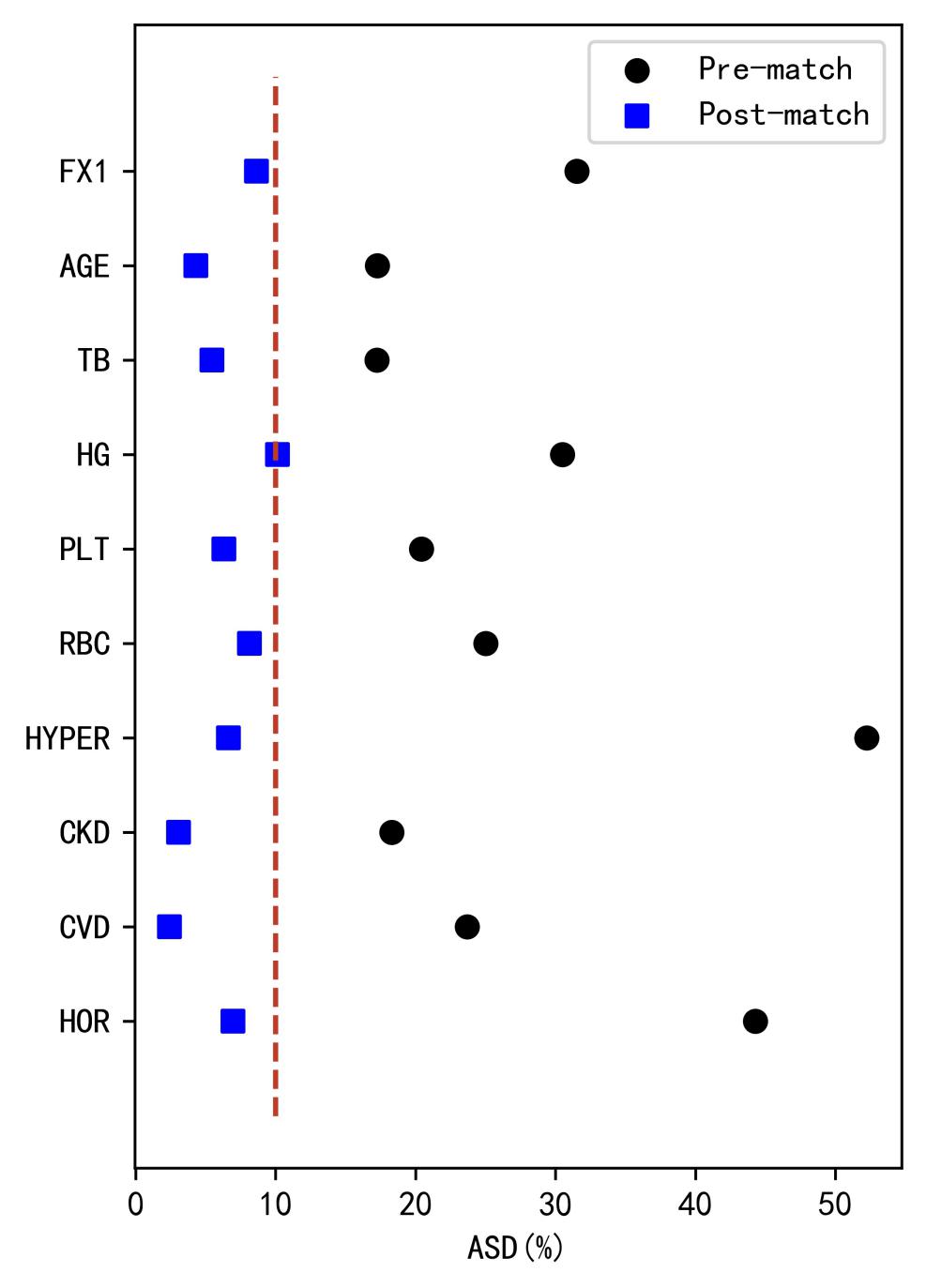
**

**Supplementary Figure 1. Standardized mean differences in unadjusted and adjusted cohorts**

**Supplementary Table 1. Missing baseline laboratory data before propensity score matching.**

| **Characteristics** | **Miss** | **Total (n=1356)** |
| --- | --- | --- |
| **Gender, n(%)** |  |  |
| Male | 0 | 470(34.66) |
| Female | 0 | 886(65.34) |
| BMI(kg/m2), mean(±SD) | 377 | 23.95±3.72 |
| Age(yr), median[IQR] | 0 | 69[59,77] |
| **Comorbidities, n(%)** |  |  |
| Diabetes mellitus | 0 | 358(26.40) |
| Cancer | 0 | 179(13.20) |
| Hypertension | 0 | 398(29.35) |
| Cardiovascular disease | 0 | 222(16.37) |
| Cerebral infarction | 0 | 133(9.81) |
| Chronic kidney disease | 0 | 216(15.93) |
| Chronic obstructive pulmonary disease | 0 | 46(3.39) |
| Chronic liver disease | 0 | 43(3.17) |
| **Clinical categories, n (%)** | 0 |  |
| Moderate |  | 390(28.76) |
| Severe |  | 548(40.41) |
| Critical |  | 418(30.83) |
| **Co-medications, n(%)** |  |  |
| Baricitinib ,n(%) | 0 | 22(1.62) |
| Systemic steroid ,n(%) | 0 | 902(66.52) |
| Tocilizumab,n(%) | 0 | 17(1.25) |
| **Laboratory maker, mean(±SD)** |  |  |
| RBC(*10^12^/L ) | 13 | 3.95±0.76 |
| WBC(*10^9^/L) | 13 | 7.60±12.59 |
| Hg(g/L) | 13 | 120.15±22.55 |
| PLT(*10^9^/L) | 13 | 213.95±229.23 |
| NEU(*10^9^/L) | 13 | 5.97±10.93 |
| NEUP(%) | 13 | 75.33±14.87 |
| AST(U/L) | 17 | 47.14±191.62 |
| ALT(U/L) | 18 | 34.66±83.35 |
| ALP(U/L) | 20 | 81.31±46.76 |
| LDH(U/L) | 18 | 318.76±176.99 |
| TB(umol/L) | 20 | 10.48±7.17 |
| eGFR(ml/min/1.73m^2^) | 20 | 71.23±28.81 |
| CCR(umol/L) | 18 | 129.17±187.37 |
| U(mmol/L) | 18 | 8.89±7.62 |
| UA(mmol/L) | 24 | 295.29±135.80 |
| NA(mmol/L) | 24 | 137.11±4.85 |
| K(mmol/L) | 24 | 4.17±0.59 |
| CL(mmol/L) | 24 | 101.61±5.17 |
| TT(s) | 55 | 18.21±9.26 |
| FBG(g/L) | 55 | 4.69±1.39 |
| APTT(s) | 54 | 37.75±9.37 |
| PT(s) | 54 | 13.53±2.32 |

**Supplementary Table 2.** **Subgroup analysis of clinical effectiveness for the composite disease progression outcome among azvudine versus nirmatrelvir-ritonavir recipients.**

| Covariates | Subgroup | N | OR | 95%CI | P-value |
| --- | --- | --- | --- | --- | --- |
| Overall |  | 725 | 1.102 | [0.743,1.634] | 0.629 |
| Gender | Male | 258 | 0.722 | [0.329,1.584] | 0.416 |
|  | Female | 467 | 1.28 | [0.806,2.031] | 0.295 |
| Diabetes mellitus | No | 548 | 0.845 | [0.531,1.345] | 0.478 |
|  | **Yes** | **177** | **2.404** | **[1.095,5.277]** | **0.029** |
| Cancer | No | 606 | 1.084 | [0.709,1.659] | 0.71 |
|  | Yes | 119 | 1.23 | [0.432,3.503] | 0.699 |
| Hypertension | No | 633 | 0.973 | [0.626,1.511] | 0.903 |
|  | Yes | 92 | 2.12 | [0.831,5.413] | 0.116 |
| Cardiovascular disease | No | 627 | 1.151 | [0.745,1.777] | 0.527 |
|  | Yes | 98 | 1.152 | [0.433,3.067] | 0.776 |
| Cerebral infarction | No | 666 | 0.973 | [0.639,1.481] | 0.899 |
|  | Yes | 59 | 3.282 | [0.950,11.334] | 0.06 |
| Chronic kidney disease | No | 577 | 1.022 | [0.654,1.596] | 0.924 |
|  | Yes | 148 | 1.451 | [0.623,3.380] | 0.388 |
| Chronic obstructive pulmonary disease | No | 704 | 1.104 | [0.741,1.644] | 0.628 |
|  | Yes | 21 | 1 | [0.075,13.367] | 1 |
| Chronic liver disease | No | 702 | 1.122 | [0.750,1.679] | 0.575 |
|  | Yes | 23 | 0.88 | [0.125,6.192] | 0.898 |
| Clinical categories | Moderate | 216 | 1.1 | [0.479,2.524] | 0.822 |
|  | Severe | 331 | 1.247 | [0.667,2.330] | 0.49 |
|  | Critical | 178 | 1.029 | [0.526,2.012] | 0.934 |
| Tocilizumab | No | 711 | 1.108 | [0.737,1.665] | 0.622 |
|  | Yes | 14 | 0.222 | [0.017,2.970] | 0.256 |
| Baricitinib | No | 709 | 1.116 | [0.747,1.666] | 0.593 |
|  | Yes | 16 | 0.556 | [0.065,4.755] | 0.592 |

OR = odds ratio, CI = confidence interval.

**Supplementary Table 3. Subgroup analysis of clinical effectiveness for the intensive care unit admission among azvudine versus nirmatrelvir-ritonavir recipients.**

| Covariates | Subgroup | N | OR | 95%CI | P-value |
| --- | --- | --- | --- | --- | --- |
| Overall |  | **725** | **2.291** | **[1.025,5.122]** | **0.043** |
| Gender | Male | 258 | 1.464 | [0.383,5.591] | 0.577 |
|  | Female | 467 | 2.973 | [1.061,8.330] | 0.038 |
| Diabetes mellitus | No | 548 | 2 | [0.834,4.797] | 0.12 |
|  | Yes | 177 | 5.238 | [0.534,51.428] | 0.155 |
| Cancer | **No** | **606** | **2.631** | **[1.106,6.258]** | **0.029** |
|  | Yes | 119 | 0.849 | [0.075,9.640] | 0.895 |
| Hypertension | No | 633 | 1.932 | [0.808,4.622] | 0.139 |
|  | Yes | 92 | 6.429 | [0.640,64.583] | 0.114 |
| Cardiovascular disease | No | 627 | 2.324 | [0.921,5.864] | 0.074 |
|  | Yes | 98 | 2.833 | [0.535,15.000] | 0.221 |
| Chronic kidney disease | No | 577 | 1.611 | [0.644,4.030] | 0.308 |
|  | **Yes** | **148** | **9.2** | **[1.046,80.937]** | **0.045** |
| Clinical categories | Moderate | 216 | 3.872 | [0.734,20.428] | 0.111 |
|  | Severe | 331 | 1.667 | [0.547,5.082] | 0.369 |
|  | Critical | 178 | 2.825 | [0.459,17.372] | 0.262 |
| Tocilizumab | No | 711 | 2.366 | [1.022,5.474] | 0.044 |
|  | Yes | 14 | 1 | [0.050,19.963] | 1 |
| Baricitinib | No | 709 | 2.37 | [1.024,5.485] | 0.044 |
|  | Yes | 16 | 1 | [0.052,19.359] | 1 |

OR = odds ratio, CI = confidence interval.

**Supplementary Table 4.** **Subgroup analysis of clinical effectiveness for the need for invasive mechanical ventilation among azvudine versus nirmatrelvir-ritonavir recipients.**

| Covariates | Subgroup | N | OR | 95%CI | P-value |
| --- | --- | --- | --- | --- | --- |
| Overall |  | **725** | **1.499** | **[1.028,2.184]** | **0.035** |
| Gender | No | 258 | 1.9 | [0.955,3.780] | 0.067 |
|  | Yes | 467 | 1.349 | [0.858,2.120] | 0.195 |
| Diabetes mellitus | No | 548 | 1.199 | [0.770,1.869] | 0.422 |
|  | **Yes** | **177** | **2.771** | **[1.321,5.812]** | **0.007** |
| Cancer | No | 606 | 1.437 | [0.954,2.163] | 0.083 |
|  | Yes | 119 | 1.912 | [0.725,5.042] | 0.19 |
| Hypertension | No | 633 | 1.408 | [0.934,2.123] | 0.102 |
|  | Yes | 92 | 2.246 | [0.852,5.921] | 0.102 |
| Cardiovascular disease | **No** | **627** | **1.823** | **[1.189,2.794]** | **0.006** |
|  | Yes | 98 | 1.018 | [0.406,2.550] | 0.969 |
| Cerebral infarction | No | 666 | 1.407 | [0.945,2.094] | 0.093 |
|  | Yes | 59 | 2.725 | [0.818,9.080] | 0.103 |
| Chronic kidney disease | No | 577 | 1.339 | [0.883,2.033] | 0.17 |
|  | Yes | 148 | 2.569 | [1.040,6.345] | 0.041 |
| Chronic obstructive pulmonary disease | **No** | **704** | **1.502** | **[1.024,2.203]** | **0.037** |
|  | Yes | 21 | 1.467 | [0.184,11.718] | 0.718 |
| Clinical categories | Moderate | **216** | **2.89** | **[1.254,6.661]** | **0.013** |
|  | Severe | 331 | 1.516 | [0.837,2.749] | 0.17 |
|  | Critical | 178 | 1.097 | [0.570,2.113] | 0.781 |
| Tocilizumab | **No** | **711** | **1.607** | **[1.093,2.364]** | **0.016** |
|  | Yes | 14 | 0.067 | [0.005,0.970] | 0.047 |
| Baricitinib | **No** | **709** | **1.509** | **[1.030,2.212]** | **0.035** |
|  | Yes | 16 | 1 | [0.104,9.614] | 1 |

OR = odds ratio, CI = confidence interval.

**Supplementary Table 5. Subgroup analysis of clinical effectiveness for the in-hospital death among azvudine versus nirmatrelvir-ritonavir recipients.**

| Covariates | Subgroup | N | OR | 95%CI | P-value |
| --- | --- | --- | --- | --- | --- |
| Overall |  | 725 | 0.997 | [0.642,1.550] | 0.991 |
| Gender | Male | 258 | 0.611 | [0.232,1.610] | 0.319 |
|  | Female | 467 | 1.144 | [0.691,1.893] | 0.602 |
| Diabetes mellitus | No | 548 | 0.696 | [0.405,1.195] | 0.189 |
|  | Yes | 177 | 2.426 | [1.057,5.570] | 0.037 |
| Cancer | No | 606 | 1.008 | [0.626,1.624] | 0.972 |
|  | Yes | 119 | 0.94 | [0.294,3.007] | 0.917 |
| Hypertension | No | 633 | 0.978 | [0.596,1.605] | 0.931 |
|  | Yes | 92 | 1.168 | [0.429,3.179] | 0.762 |
| Cardiovascular disease | No | 627 | 1.017 | [0.621,1.667] | 0.945 |
|  | Yes | 98 | 1.203 | [0.432,3.353] | 0.724 |
| Cerebral infarction | No | 666 | 0.853 | [0.529,1.374] | 0.513 |
|  | Yes | 59 | 3.3 | [0.893,12.192] | 0.073 |
| Chronic kidney disease | No | 577 | 0.922 | [0.559,1.522] | 0.751 |
|  | Yes | 148 | 1.321 | [0.517,3.371] | 0.561 |
| Chronic obstructive pulmonary disease | No | 704 | 0.998 | [0.638,1.560] | 0.992 |
|  | Yes | 21 | 1 | [0.075,13.367] | 1 |
| Chronic liver disease | No | 702 | 1.017 | [0.646,1.602] | 0.941 |
|  | Yes | 23 | 0.88 | [0.125,6.192] | 0.898 |
| Clinical categories | Moderate | 216 | 0.661 | [0.241,1.811] | 0.421 |
|  | Severe | 331 | 1.278 | [0.638,2.558] | 0.489 |
|  | Critical | 178 | 1.019 | [0.495,2.098] | 0.96 |
| Tocilizumab | No | 711 | 0.967 | [0.614,1.525] | 0.887 |
|  | Yes | 14 | 1 | [0.120,8.306] | 1 |
| Baricitinib | No | 709 | 0.984 | [0.627,1.544] | 0.945 |
|  | Yes | 16 | 1 | [0.104,9.614] | 1 |

OR = odds ratio, CI = confidence interval.

**Supplementary Table 6. Subgroup analysis of clinical effectiveness for all ADR events** **among azvudine versus nirmatrelvir-ritonavir recipients.**

| Covariates | Subgroup | N | OR | 95%CI | P-value |
| --- | --- | --- | --- | --- | --- |
| Overall |  | 725 | 1.179 | [0.672,2.068] | 0.566 |
| Gender | Male | 258 | 1.276 | [0.524,3.110] | 0.592 |
|  | Female | 467 | 1.124 | [0.544,2.321] | 0.752 |
| Diabetes mellitus | No | 548 | 1.335 | [0.695,2.565] | 0.385 |
|  | Yes | 177 | 0.828 | [0.270,2.536] | 0.741 |
| Hypertension | No | 633 | 1.408 | [0.764,2.596] | 0.272 |
|  | Yes | 92 | 0.457 | [0.091,2.296] | 0.342 |
| Cardiovascular disease | No | 627 | 1.542 | [0.822,2.890] | 0.177 |
|  | Yes | 98 | 0.436 | [0.090,2.113] | 0.303 |
| Cerebral infarction | No | 666 | 1.149 | [0.610,2.163] | 0.668 |
|  | Yes | 59 | 1.384 | [0.378,5.061] | 0.623 |
| Chronic kidney disease | No | 577 | 1.144 | [0.617,2.123] | 0.669 |
|  | Yes | 148 | 1.38 | [0.355,5.374] | 0.642 |
| Clinical categories | Moderate | 216 | 0.881 | [0.308,2.521] | 0.814 |
|  | Severe | 331 | 1.342 | [0.601,2.997] | 0.473 |
|  | Critical | 178 | 1.33 | [0.404,4.377] | 0.639 |
| Systemic steroid | No | 137 | 2.047 | [0.396,10.564] | 0.392 |
|  | Yes | 588 | 1.082 | [0.593,1.973] | 0.797 |

OR = odds ratio, CI = confidence interval.
